# Traditional Chinese Medicine studies for Alzheimer’s disease via network pharmacology based on entropy and random walk

**DOI:** 10.1371/journal.pone.0294772

**Published:** 2023-11-29

**Authors:** Xiaolu Wu, Shujuan Cao, Yongming Zou, Fangxiang Wu

**Affiliations:** 1 School of Mathematical Sciences, Tiangong University, Tianjin, China; 2 Department of Neurology, Tianjin Huanhu Hospital, Tianjin, China; 3 Division of Biomedical Engineering, Department of Mechanical Engineering and Department of Computer Science, University of Saskatchewan, Saskatoon, Saskatchewan, Canada; Anhui University of Chinese Medicine, CHINA

## Abstract

Alzheimer’s disease (AD) is a common neurodegenerative disease having complex pathogenesis, approved drugs can only alleviate symptoms of AD for a period of time. Traditional Chinese medicine (TCM) contains multiple active ingredients that can act on multiple targets simultaneously. In this paper, a novel algorithm based on entropy and random walk with the restart of heterogeneous network (RWRHE) is proposed for predicting active ingredients for AD and screening out the effective TCMs for AD. First, Six TCM compounds containing 20 herbs from the AD drug reviews in the CNKI (China National Knowledge Internet) are collected, their active ingredients and targets are retrieved from different databases. Then, comprehensive similarity networks of active ingredients and targets are constructed based on different aspects and entropy weight, respectively. A comprehensive heterogeneous network is constructed by integrating the known active ingredient-target association information and two comprehensive similarity networks. Subsequently, bi-random walks are applied on the heterogeneous network to predict active ingredient-target associations. AD related targets are selected as the seed nodes, a random walk is carried out on the target similarity network to predict the AD-target associations, and the associations of AD-active ingredients are inferred and scored. The effective herbs and compounds for AD are screened out based on their active ingredients’ scores. The results measured by machine learning and bioinformatics show that the RWRHE algorithm achieves better prediction accuracy, the top 15 active ingredients may act as multi-target agents in the prevention and treatment of AD, Danshen, Gouteng and Chaihu are recommended as effective TCMs for AD, Yiqitongyutang is recommended as effective compound for AD.

## 1. Introduction

Alzheimer’s disease (AD) is a persistent and irreversible neurodegenerative disease, whose main clinical features are progressive memory loss, cognitive declination, functional independence loss and so on [1]. The pathogenesis of AD is complex, and not fully understood yet. The incidence of AD has been increasing in recent years, which brings a heavy economic burden to the healthcare system around the world. However, current clinical AD drugs approved by the U.S. Food and Drug Administration (FDA) only relieve related symptoms within a certain period, but none of these therapies can effectively halt the development of the disease. Other single-target AD drugs have failed in clinical trials. Therefore, it is essential to identify efficient and low-toxicity AD drugs with multi-targets [2].

Traditional Chinese medicine (TCM) contains multiple active ingredients that can act on multi-targets simultaneously, some active pharmacological compounds of herbs have been proven to be applied to the treatment of many diseases [3]. TCM has a long history of AD, many active ingredients extracted from herbs have fewer side effects and are regarded as potential anti-AD drugs [4]. However, the potential molecular mechanism of TCM for AD is still unclear, which limits further clinical applications. Network pharmacology is integral and systematic by integrating the ideas of pharmacology with network science, systems biology, and bioinformatics, which can be used to screen active ingredients and understand the mechanism of multi-ingredient, multi-target, and multi-pathway of TCM [5]. Therefore, network pharmacology can be used to reveal the associations between AD, active ingredients and targets, which open up a new way to study the mechanism between TCMs and AD.

With the development of high-throughput biomedical data, network-based propagation methods are often used to predict associations among biological components. Random Walk is one of the typical methods based on network propagation. The traditional restart random walk (RWR) algorithm only carries out random walks in a single network [6], many scholars have proposed improved algorithms for RWR on heterogeneous networks. Luo et al. proposed a random walk-based algorithm on the Reliable Heterogeneous Network (RWRHN) to prioritize potential candidate genes for inherited diseases [7]. Li et al. presented a superimposed local random walk algorithm called LRWHLDA to predict the associations between LncRNAs and diseases. Their algorithm overcomes the limitation of lack of known association between nodes [8]. The topological and structural properties of different networks are different. Wang et al. quantified the individual walk length of nodes by using the improved Jaccard index, and proposed an individual bi-random walk algorithm called DR-IBRW for drug repositioning [9]. Luo et al. put forward a bi-random walk algorithm (MBIRW) for drug repositioning, random walks are conducted to predict potential drug-disease associations in the drug similarity network and disease similarity network respectively [10]. The algorithm based on network inference is also applied to the prediction of biological information association. Cheng et al. proposed an algorithm based on network-based inference (NBI), known drug-target association information was used as the initial resource allocation, the final resource allocation information was obtained through two-step propagation to predict drug-target associations [11]. Wang et al. proposed a method based on the guilt-by-association principle, called HGBI for prediction of novel drug-target associations [12]. In addition, KATZ is also a network-based algorithm. Zhu et al. considered the contribution of different walk lengths to the similarity network, and proposed the HMDAKATZ algorithm to predict bacteria-drugs associations [13].

In this paper, we develop a novel algorithm based on entropy and random walk with the restart of heterogeneous network (RWRHE) for predicting active ingredients associated with AD and screening out the effective TCMs for AD. Firstly, the similarity measures of active ingredients are calculated from two aspects, including the chemical structure and the Gaussian interaction profile kernel (GIP kernel) similarity [14]. The similarity measures of targets are calculated from four aspects, including protein sequence, interaction score in String database(release 2021–08) [15], common neighbor, and GIP kernel similarity. Secondly, based on the weight of each similarity measure assigned by information entropy, similarity measures of active ingredients and targets are integrated into comprehensive similarity measures, respectively. Therefore, a heterogeneous network can be constructed by connecting the comprehensive active ingredient network and target network via known active ingredient-target associations. Next, the active ingredients-target association score matrix is calculated by bi-random walk on heterogeneous networks. Then, AD related targets are selected as the seed nodes, and the random walk is performed on the target similarity network to calculate the AD-target association score vector. Finally, the relationships between AD and active ingredients are predicted and scored, the effects of TCMs for AD are scored and ranked. Gene ontology (GO) and Kyoto Encyclopedia of Genes and Genomes (KEGG) enrichment analysis [16] are performed on the top 100 targets by different methods to illustrate the relationship between topological properties and relevant biological functions. Molecular docking is used to assess the ability of top 15 the active ingredients to enter the active pocket of the key enzymes or proteins of AD, the results show that the active ingredients may act as multi-target agents in the prevention and treatment of AD.

## 2. Materials and methods

### 2.1. Datasets

Six TCM compounds from the AD drug reviews in the CNKI (China National Knowledge Internet) are selected, including Dangguishaoyaosan, Yigansan, Yiqitongyutang, Gubenjiannaoye, BKHJ TCM and Yizhi [17–20]. A total of 20 herbs are involved. The active ingredients of these herbs are retrieved from Traditional Chinese Medicine System Pharmacology (TCMSP) database (release 2014–05) [21]. Based on the condition that oral bioavailability (OB) is more than 20% and drug-likeness (DL) is more than 0.1, 583 active ingredients are screened out. The herbs contained in TCM compounds and the active ingredients contained in the herbs are shown in S1 Table.

The datasets used in this study include active ingredients, targets and known active ingredient-target associations, which are collected from two databases: TCMSP and HERB (release 2021–01) [22]. Targets associated with active ingredients are mapped into genes through UniProt database (release 2022–05) [23] for “Homo sapiens” organism. 4313 active ingredient-target associations involving 387 active ingredients and 374 targets are retrieved from TCMSP database. Simultaneously, 5823 active ingredient-target associations involving 453 active ingredients and 642 targets are obtained from HERB database. We take the union of these two datasets as the total dataset, which has 6973 active ingredient-target associations involving 457 active ingredients and 731 targets, as shown in S2 Table.

### 2.2 Similarity measures

#### 2.2.1 Active ingredient similarity measures

Let $R=\left\{r_{1},r_{2},\cdots ,r_{n_{r}}\right\}$ be the set of *n*_*r*_ active ingredients and $T=\left\{t_{1},t_{2},\cdots ,t_{n_{t}}\right\}$ be the set of *n*_*t*_ targets. The matrix *A*_*rt*_ represents known active ingredient-target associations, and its dimension is *n*_*r*_ * *n*_*t*_. The value of *A*_*rt*_(*i*, *j*) is 1 if active ingredient *i* and target *j* have a known association, otherwise is 0.

The first similarity measure is calculated based on the chemical structure. The SMILES (Simplified Molecular Input Line Entry Specification) describes the chemical structure of active ingredients [24], which could be retrieved from PubChem database (release 2022–06) [25]. For the active ingredients that cannot be retrieved, the SMILES can be obtained from their 2D structure through the Swiss Target Prediction database (release 2019–05) [26]. The Chemical Development Kit is used to compute chemical fingerprints of active ingredients [27]. The similarity between active ingredients *r*_*i*_ and *r*_*j*_ is calculated by the Tanimoto score of the binary fingerprint vector [28], the formula as follows:

$$S_{r}^{f}(r_{i},r_{j})=\frac{f_{r_{i}}*f_{r_{j}}}{f_{r_{i}}^{2}+f_{r_{i}}^{2}-f_{r_{i}}*f_{r_{j}}}$$ (1)

where $f_{r_{i}}$ represents the chemical fingerprint vector of the active ingredient *r*_*i*_.

Based on the finding that weak similarity between active ingredient pairs provides less information for prediction, the logistic function is used to convert those small similarity values into values close to zero and expand those large similarity values simultaneously [10]. The improved similarity value by the logistic function is redefined as follows:

$$L(S_{r}^{f}(r_{i},r_{j}))=\frac{1}{1+\text{exp}(c\cdot S_{r}^{f}(r_{i},r_{j})+d)}$$ (2)

where *c* and *d* are the parameters. According to the previous research [29, 30], we set *d* as log(9999), which represents *L*(0) = 0.0001. We set *L*(0.3) < 0.01, which determines *c* as -15. The improved similarity value $L\left(S_{r}^{f}\left(r_{i},r_{j}\right)\right)$ is denoted as $S_{r}^{1}\left(r_{i},r_{j}\right)$.

Based on the assumption that similar active ingredients tend to associate with similar targets, GIP kernel similarity is used to calculate the similarity between active ingredients. The interaction profile *IP*(*r*_*i*_) of active ingredient *r*_*i*_ is a binary vector representing the known associations between the active ingredient *r*_*i*_ and targets. The GIP kernel similarity between active ingredient *r*_*i*_ and active ingredient *r*_*j*_ is computed as follows:

$$\begin{array}{l} S_{r}^{2}(r_{i},r_{j})=exp(-{ϒ}_{r}{\left\|IP(r_{i})-IP(r_{j})\right\|}^{2})\\ {ϒ}_{r}={ϒ}_{r}{}^{\prime }/\left(\frac{1}{n_{r}}{\sum_{i=1}^{n_{r}}\left\|IP(r_{i})\right\|}^{2}\right) \end{array}$$ (3)

where *IP*(*r*_*i*_) denotes the i-th row of the matrix *A*_*rt*_. $\Upsilon_{r}^{\prime }$ is set to be 1 according to previous research [14]. The similarity values of active ingredients based on the above two similarity measures are shown in S3 Table.

#### 2.2.2 Target similarity measures

The first target similarity measure is calculated based on the protein sequences, which can be retrieved from UniProt database. The Smith-Waterman local alignment algorithm [31] is used to calculate the sequence similarity of targets, and the similarity matrix is denoted as $S_{t}^{1}$.

The second target similarity measure is based on the interaction confidence scores of the String database. The gene symbols of targets are entered into the String database, and the organisms are selected as "Homo sapiens". Then the interaction scores of target pairs are as elements of the second similarity matrix $S_{t}^{2}$.

The third target similarity measure is calculated based on the common neighbors of targets. To filter out the target interaction relationships with low confidence, target pairs with interaction scores greater than 0.4 in the String database are regarded as neighbors. The contribution of the common neighbor targets with small degree is greater than that of the common neighbor targets with large degree, each common neighbor target is assigned a weight based on its degree value. The target similarity measure based on the common neighbors is defined as:

$$S_{t}^{3}(t_{i},t_{j})=\sum_{z\in \Gamma (t_{i})\cap \Gamma (t_{j})}\frac{1}{k(z)}$$ (4)

where Γ(*t*_*i*_) is the set of neighbors of target *t*_*i*_, *z* are the common neighbors of targets *t*_*i*_ and *t*_*j*_, *k*(*z*) is the degree of *z*. If two targets have more common neighbors and the degree of common neighbors is small, the similarity between them will be greater than 1, it will be replaced by 0.99.

Based on the assumption that similar targets tend to associate with similar active ingredients, the fourth target similarity measure based on GIP kernel is defined. The interaction profile *IP*(*t*_*i*_) of target *t*_*i*_ is a binary vector representing the known associations between target *t*_*i*_ and active ingredients. The GIP kernel similarity between targets *t*_*i*_ and *t*_*j*_ is computed as follows:

$$\begin{array}{l} S_{t}^{4}(t_{i},t_{j})=exp(-{ϒ}_{t}{\left\|IP(t_{i})-IP(t_{j})\right\|}^{2})\\ {ϒ}_{t}={ϒ}_{t}{}^{\prime }/\left(\frac{1}{n_{t}}{\sum_{i=1}^{n_{t}}\left\|IP(t_{i})\right\|}^{2}\right) \end{array}$$ (5)

where *IP*(*t*_*i*_) denotes the i-th column of the matrix *A*_*rt*_. $\Upsilon_{t}^{\prime }$ is set to be 1 according to previous research [14]. The similarity values of targets based on the above four similarity measures are shown in S4 Table.

#### 2.2.3 Integrating similarity measures based on entropy

Different similarity measures contain different similarity information and play different roles in measuring the similarity of node pairs. The information entropy is used to select similarity measures in the previous research [32]. In this study, the weights of different similarity measures are further calculated based on entropy.

For the m-th similarity matrix for active ingredients, the entropy $E_{i}^{m}$ of the i-th row is calculated as follows:

$$E_{i}^{m}=-\sum_{j=1}^{k}\frac{s_{ij}}{\sum_{j=1}^{k}s_{ij}}\text{log}\left(\frac{s_{ij}}{\sum_{j=1}^{k}s_{ij}}\right)$$ (6)

where *s*_*ij*_ represents the similarity value between active ingredients *i* and *j*. *k* indicates the number of active ingredients. We average the entropy of all rows as the final average entropy value. The average entropy of the m-th similarity matrix is calculated as follows:

Em=mean∑i=1kEik (7)

The smaller the average entropy of the similarity matrix is, the less random information is delivered by the similarity measure. The similarity measure with small average entropy occupies a significant proportion of the comprehensive similarity measure. The average entropy of each similarity matrix is normalized after taking the reciprocal, then the weight of the m-th similarity measure is defined as follows:

$$\omega_{rm}=\frac{1/E_{mean}^{m}}{\sum_{n}1/E_{mean}^{n}}$$ (8)

where *ω*_*rm*_ represents the weight of the m-th similarity measure of active ingredients. In the same way, the average entropy and weight of each target similarity measure can be obtained. The average entropy and corresponding weights of the similarity matrix of two active ingredients and four targets are shown in Table 1.

**Table 1:** Entropy and weight of similarity measure.

		*E*_*mean*_	*ω*
Active ingredient	$S_{r}^{1}$	3.9734	0.5986
	$S_{r}^{2}$	5.9265	0.4014
Target	$S_{t}^{1}$	6.5938	0.2071
	$S_{t}^{2}$	4.5929	0.2974
	$S_{t}^{3}$	4.9661	0.2750
	$S_{t}^{4}$	6.1959	0.2204

Finally, the two similarity measures of active ingredients are integrated into a comprehensive active ingredient similarity measure *S*_*r*_, and the four similarity measures of targets are integrated into a comprehensive target similarity measure *S*_*t*_, which are calculated as follows:

$$\begin{array}{c} S_{r}=\omega_{r1}S_{r}^{1}+\omega_{r2}S_{r}^{2}\\ S_{t}=\omega_{t1}S_{t}^{1}+\omega_{t2}S_{t}^{2}+\omega_{t3}S_{t}^{3}+\omega_{t4}S_{t}^{4} \end{array}$$ (9)

where *ω*_*r*1_ and *ω*_*r*2_ are the weights of active ingredient similarity matrix $S_{r}^{1}$ and $S_{r}^{2}$, respectively. *ω*_*t*1_, *ω*_*t*2_, *ω*_*t*3_, *ω*_*t*4_ are the weights of target similarity matrix $S_{t}^{1},S_{t}^{2},S_{t}^{3},S_{t}^{4}$, respectively. The comprehensive similarity values of active ingredients and targets are shown in S5 Table.

### 2.3 Construction of the heterogeneous network

The similarity networks of active ingredients and targets are constructed based on the comprehensive similarity measures of active ingredients and targets. $R=\left\{r_{1},r_{2},\cdots ,r_{n_{r}}\right\}$ is the node set of *n*_*r*_ active ingredients. The comprehensive similarity *S*_*r*_(*r*_*i*_, *r*_*j*_) is the weight between active ingredients *r*_*i*_ and *r*_*j*_. $T=\left\{t_{1},t_{2},\cdots ,t_{n_{t}}\right\}$ is the node set of *n*_*t*_ targets. The comprehensive similarity *S*_*t*_(*t*_*i*_, *t*_*j*_) is the weight between targets *t*_*i*_ and *t*_*j*_.

In addition, the active ingredient-target bipartite graph *G*(*V*, *E*) is constructed based on the known active ingredient-target associations. *V* = {*R*, *T*} is the node set containing active ingredient nodes and target nodes. *E* = {*A*_*rt*_(*i*,*j*)} is the edge set. If active ingredient *i* and target *j* have a known association, the weight of edge between them is 1, otherwise is 0.

**Fig 1:**
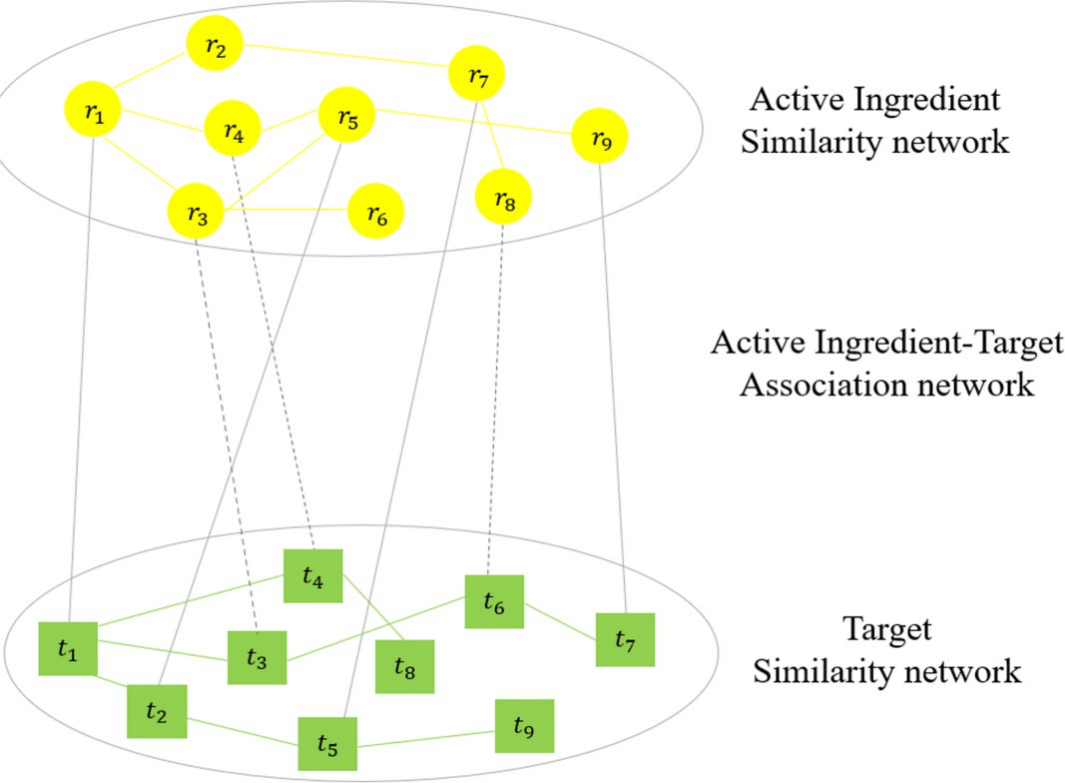
The active ingredient-target heterogeneous network. Yellow circles and green rectangles represent active ingredients and targets respectively.

The active ingredient-target heterogeneous network can be constructed by integrating the active ingredient similarity network, target similarity network, and the known active ingredient-target association network. The heterogeneous network is illustrated in Fig 1. The yellow circles and green rectangles represent active ingredients and targets, respectively. Solid lines represent known active ingredient-target associations, dashed lines indicate the predicted active ingredient-target associations.

### 2.4 Bi-random walk on the heterogeneous network

On the heterogeneous network, a bi-random walk algorithm is used to predict the active ingredient-target associations score matrix. As the previous research [33], the Laplacian normalization is used to normalize the weight matrix of a network. For the active ingredient similarity matrix *S*_*r*_, the Laplacian normalization is divided into two steps:

$$S_{r}^{\prime }(r_{i},r_{j})=\frac{S_{r}(r_{i},r_{j})}{\sqrt{D(r_{i},r_{i})*D(r_{j},r_{j})}}$$ (10)

$$S_{r}^{\prime \prime }(r_{i},r_{j})=\frac{S_{r}^{\prime }(r_{i},r_{j})}{\sum_{j}S_{r}^{\prime }(r_{i},r_{j})}$$ (11)

where *D* is a diagonal matrix, and the elements of the main diagonal are the sum of corresponding rows of the matrix *S*_*r*_, i.e $D(r_{i},r_{i})=\sum_{k=1}^{n_{r}}S_{r}(r_{i},r_{k})$. The matrix *S*_*r*_ after Laplacian normalization is denoted as $S_{r}^{\prime \prime }$. Likewise, the target similarity matrix *S*_*t*_ after Laplacian normalization is denoted as $S_{t}^{\prime \prime }$. In addition, the adjacency matrix *A*_*rt*_ of the active ingredient-target associations is normalized as follows:

$$A_{rt}^{\prime }=\frac{A_{\text{rt}}}{sum(A_{\text{rt}})}$$ (12)

A random walk begins from an active ingredient, then traverses to other target nodes based on its associated targets. The probabilistic associations between the active ingredient and all targets are obtained. The left random walk is performed on the target similarity network to simulate this process:

$$leftRT_{t}=\alpha \times RT_{t-1}*S_{t}^{\prime \prime }+(1-\alpha )\times A_{rt}^{\prime }$$ (13)

where *α* = 0.3 is the restart probability, which controls the probability for the walker staying at the starting node. *leftRT*_*t*_ is the predicted association matrix between active ingredients and targets in the t-step iteration, $RT_{0}=A_{rt}^{\prime }$. The left random walk stops until |*leftRT*_*t*+1_ − *leftRT*_*t*_| < 10^−6^, the resulted matrix is denoted *leftRT*.

Likewise, a random walk starts from a target node, then traverses other active ingredient nodes based on its known associated active ingredients. Then another active ingredient-target association matrix is obtained. The right random walk is conducted on the active ingredient similarity network to mimic this process:

$$rightRT_{t}=\alpha \times S_{r}^{\prime \prime }\times RT_{t-1}+(1-\alpha )\times A_{rt}^{\prime }$$ (14)

where *α* = 0.3 is the restart probability, the right random walk stops until |*rightRT*_*t*+1_ − *rightRT*_*t*_| < 10^−6^, the resulted matrix is denoted *rightRT*. The final result is the average of the left and right random walk results, and the final predicted active ingredient-target association matrix is denoted as *RT* as follows:

$$RT=\frac{leftRT+rightRT}{2}$$ (15)

The final matrix *RT* is shown in S6 Table.

### 2.5 Prediction of AD-target association

In this study, the targets from the Alzheimer’s disease pathway (hsa05010) in the KEGG and the targets contained in the AD mini metabolic network [34] are selected. The intersection of these targets and aforementioned 731 targets contains 72 targets, which form the seed node set. The initial probability vector *p*_0_ of targets is constructed such that equal probability is assigned to each target seed, and probability 0 is assigned to other targets, with the sum of the probabilities equal to 1. Starting from the target seed node, according to the topological property of the target similarity network, random walks are carried out to traverse other targets. Then, the AD-targets association probabilities vector could be predicted as follows:

$$P_{t}=\alpha *S_{t}^{\prime \prime }*P_{t-1}+(1-\alpha )P_{0}$$ (16)

where *α* = 0.3 is the restart probability. *P*_*t*_ is the probability vector of the t-step iteration, and its value represents the association probabilities between targets and AD. The iteration stops until |*P*_*t*+1_ − *P*_*t*_| < 10^−6^, the resulted probability vector is denoted as *P*, and its values are as shown in S7 Table.

### 2.6 Prediction of AD-active ingredient association

Combined with the final predicted active ingredient-target association matrix *RT* and the AD-targets association vector *P*, the association scores of active ingredients and AD are predicted as follows:

$$P_{r}(r_{i})=\sum_{j}RT(r_{i},t_{j})*P(t_{j})$$ (17)

where *RT*(*r*_*i*_, *t*_*j*_) represents the final predicted association score of active ingredient *r*_*i*_ and target *t*_*j*_, *P*(*t*_*j*_) represents the score of the target *t*_*j*_ associated with AD. *P*_*r*_(*r*_*i*_) represents the score of the active ingredient *r*_*i*_ associated with AD, as shown in S8 Table.

## 3. Results and discussion

### 3.1 Measuring the effect of similarity by ablation analysis

Different similarity measures in a network can lead to differences in the topology and structure characteristics of the network. Therefore, the iterative process of random walk and the accuracy of prediction results are affected by different measures. Ablation analysis is performed on the comprehensive similarity measure to study the influence of different similarity measures on the performance of the RWRHE algorithm. We implement two simplified variants of RWRHE.

1. RWRHE_GIP: By removing GIP kernel similarity, the active ingredient similarity $S_{r}^{2}$ and target similarity $S_{t}^{4}$ based on GIP kernel similarity are removed.
2. RWRHE_E: By removing entropy, each similarity measure is weighted equally.

**Fig 2:**
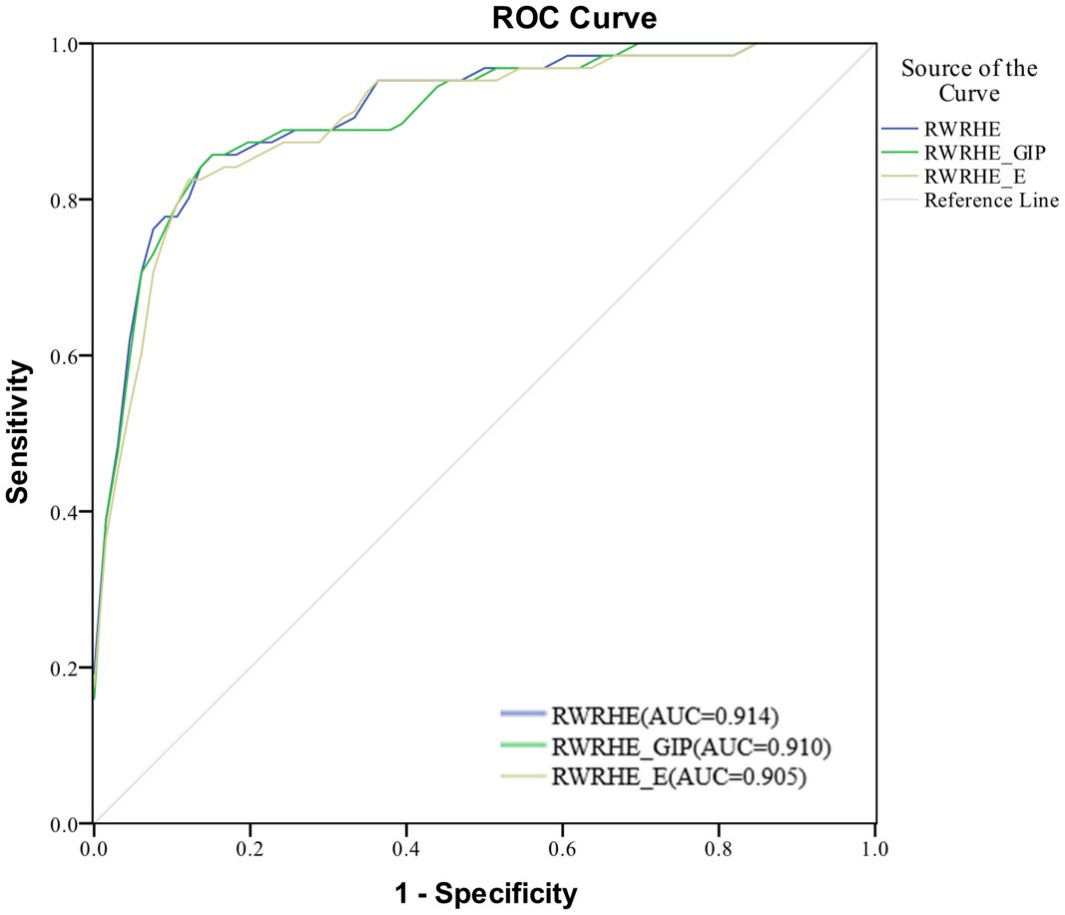
The ROC curves of RWRHE, RWRHE_GIP and RWRHE_E.

The Receiver Operating Characteristic Curve (ROC) reflects the relationship between True Positive Rate (TPR) and False Positive Rate (FPR) at different thresholds. The area under the ROC curve (AUC) is used as an evaluation metric to measure the accuracy of prediction results. “Alzheimer’s Disease” is used as the keyword, 63 active ingredients associated with AD are retrieved from the HERB database as the positive control group. At the same time, the active ingredients related to leukemia, mammary carcinoma, fever, and acute pharyngitis are retrieved, and the active ingredients related to AD are excluded, 66 active ingredients are obtained as the negative control group. The positive and negative control groups are shown in S9 Table. TPR, also known as sensitivity, represents the ratio of active ingredients in positive controls that rank for association with AD above the specified threshold. FPR, also known as 1-specificity, denotes the percentage of active ingredients in negative control group that rank for association with AD above the specified threshold. ROC curves of RWRHE, RWRHE_GIP, and RWRHE_E algorithms are shown in Fig 2. The results show that the RWRHE algorithm achieves higher prediction accuracy, and its AUC value is 0.914. The AUC values of RWRHE_GIP and RWRHE_E algorithms are 0.910 and 0.905, respectively. The number of nodes and edges in the network corresponding to the RWRHE_GIP and the RWRHE_E is the same as that of the RWRHE, but each edge has different weight. The GIP kernel similarity integrates the known active ingredient-target associations information, which makes the prediction results more reliable. The importance of each similarity measure is quantitatively weighted based on entropy, which makes the similarity network more complete. Therefore, integrating GIP kernel similarity and information entropy can improve the performance of prediction results to a certain extent.

### 3.2 Comparison with other methods

**Fig 3:**
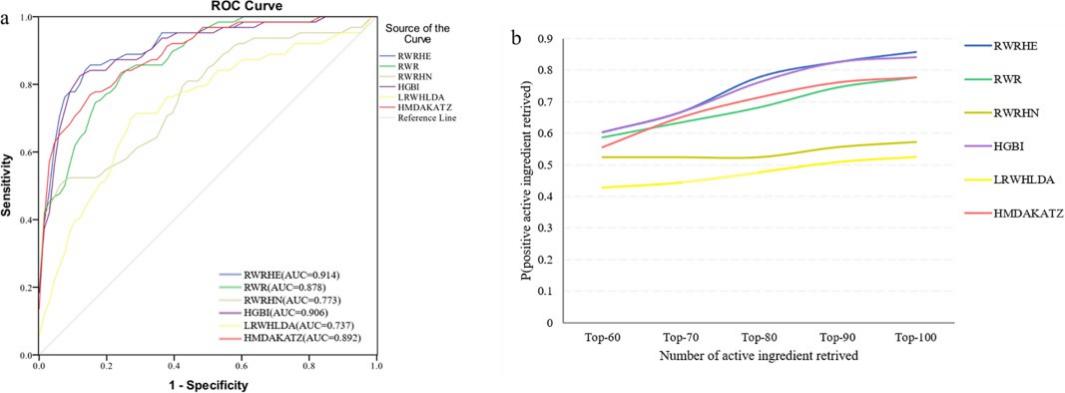
(a)The ROC curves of RWRHE, RWR, RWRHN, HGBI, HMDAKATZ and LRWHLDA. (b)The ranks of CDF.

To further evaluate the performance of RWRHE algorithm, other five network-based algorithms RWR [6], RWRHN [7], HGBI [12], LRWHLDA [8] and HMDAKATZ [13] are compared with RWRHE. RWR algorithm is implemented on the node similarity network. The walker returns to the seed node with a certain probability in the iterative process. All nodes are ranked according to the final probability. The RWRHN algorithm takes into account the jump probability of nodes in heterogeneous networks when constructing the transition matrix. Active ingredients related to AD are retrieved from the SymMap database(release 2019–01) [35], and the top 50 active ingredients in the inferred evidence score are selected as seed nodes. The RWR and RWRHN algorithms are used to calculate the association scores between AD and all active ingredients. The HGBI algorithm based on heterogeneous graph reasoning infers potential node associations by constructing heterogeneous networks. The LRWHLDA algorithm is a superimposed local random walk algorithm. The HMDAKATZ algorithm considers the contribution of different walks to the association probability, and considers that the longer walks tend to contribute less to similarity. We apply HGBI, LRWHLDA and HMDAKATZ algorithms to calculate the active ingredient-target association probability matrix, which is compared with the bi-random walk part in this study. The ROC curves of RWRHE and the other five algorithms are shown in Fig 3(a). The results show that the RWRHE algorithm has excellent prediction performance, and its AUC value is 0.914, which is higher than the AUC values of the other five algorithms.

In addition, the ranks of prediction results also play an important role in evaluating the performance of all algorithms. The cumulative distribution function (CDF) of the ranks is employed to compare the performance of different algorithms. CDF refers to the proportion of active ingredients whose ranking exceeds the top-r threshold in the positive control group. 60 ≤ *r* ≤ 100 are reported as shown in Fig 3(b). The results show that compared with the other five algorithms, the RWRHE algorithm retrieves the largest proportion under the same top-r threshold. The majority of the 63 active ingredients in the positive control group are retrieved in the top 100. Therefore, RWRHE has a good performance in predicting the relationship between AD and active ingredients.

The RWRHE algorithm predicts the association scores and ranks between active ingredients and AD. According to the active ingredients contained in herbs and ranks of active ingredients associated with AD, the effect score of herb *k* in treating AD is calculated based on ranks as follows:

$$H_{k}=\sum_{i}\frac{1}{rank(i)}$$ (18)

where, *rank*(*i*) represents the rank of the active ingredient *i* associated with AD. Finally, 20 herbs are scored and ranked according to the predicted effect scores of herbs for AD, shown in S10 Table, the results show that Danshen have the best effect for AD, followed by Gouteng and Chaihu. Similarly, based on the active ingredients contained in the compound, the efficacy of the compound for AD is scored and ranked according to Eq (18), the results are shown in Table 2. The results show that Yiqitongyutang is the best compound for AD compared with other compounds, which indicates that Yiqitongyutang may have better therapeutic effect for AD.

**Table 2:** Ranking of efficacy of compound in treating AD.

Compound	score	rank
Yiqitongyutang	6.229121413	1
Yigansan	4.196828293	2
BKHJ TCM	2.394613178	3
Gubenjiannaoye	2.28103394	4
Dangguishaoyaosan	0.991858956	5
Yizhi	0.165683049	6

### 3.3 GO enrichment analysis and KEGG pathway analysis

**Fig 4:**
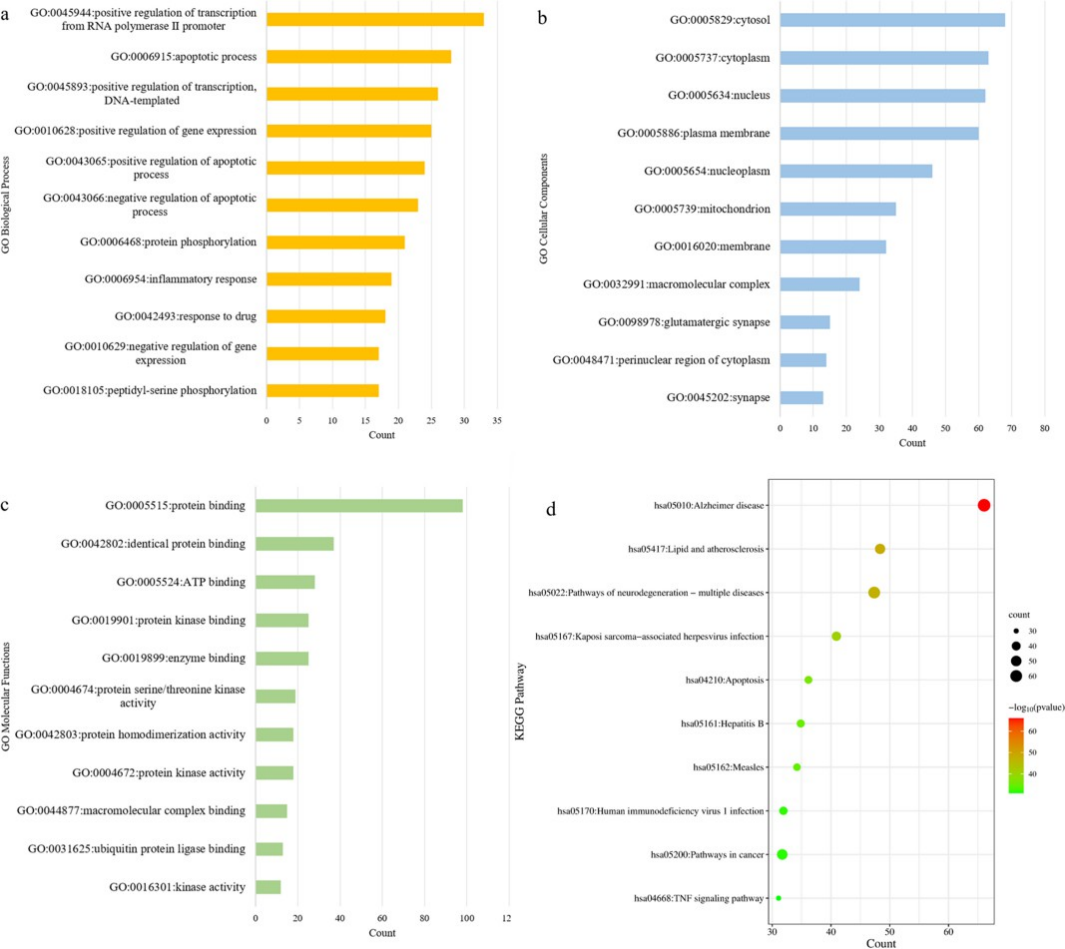
GO enrichment analysis and KEGG pathway analysis of potential targets. (a): the histogram of GO biological process; (b) the histogram of GO cell component; (c) the histogram of molecular function; (d) the bubble graph of KEGG pathway.

The top 100 targets associated with AD are regarded as potential targets of AD. To learn more information about TCMs and AD, bioinformatics analysis is used to find relevant information about potential targets of AD. GO enrichment analysis and KEGG pathway analysis are performed to clarify relevant biological information about the potential targets of AD. GO enrichment analysis includes biological process (BP), cell component (CC), and molecular function (MF). To screen out the most significantly enriched biological annotations, the top 11 entries with the lowest P value are selected respectively, as shown in Fig 4(a)–4(c). In biological process results, the most enriched GO term ‘Positive regulation of transcription from RNA polymerase II promoter’ may be involved in the transcription of the substance related to synaptic connectivity. Synapses play a central role in learning and memory, and disorders of these behaviors can lead to neurological diseases, including AD [36]. The targets are also mainly concentrated on positive and negative regulation of gene expression and apoptotic biological processes, suggesting that apoptosis and gene expression regulation play an important role in the mechanism of AD. In addition, the targets are involved in the biological process of protein phosphorylation. Protein phosphorylation is the key mechanism of AD [37]. Highly phosphorylated tau protein forms neurofibrillary tangles (NFTs), which is one of the main histopathological features of AD [38]. In cell component results, the targets are mainly concentrated on cytoplasm, nucleus, plasma membrane and so on. In molecular functional results, the targets are mainly concentrated on protein kinase binding, protein kinase activity, protein serine/threonine kinase activity and protein kinase activity. Some researchers report that Glycogen synthase kinase-3β (Gsk3β), Ccyclin-dependent kinase 5 (CDK5) and Microtubule affinity regulating kinase (MARK) may hyper-phosphorylate tau and accelerate the formation of NFTs [39–41]. Cyclin-dependent kinases (Cdks), which are serine/threonine kinases, regulate cell cycle and neuronal differentiation. Cdks pathway may have effects on neuron loss, which is responsible for AD [42].

The involvement of pathways based on potential targets by KEGG pathway enrichment analysis is plentiful. The top 10 pathways with the smallest P value are drawn as bubble graphs in Fig 4(d). The size of the dots in the bubble diagram represents the number of targets enriched in the pathway, and the depth of the color represents the size of the statistical test P-value. The number of these targets enriched in the AD pathway is the highest. In addition, the potential targets are mainly concentrated on neurodegenerative diseases, apoptosis, and cancer pathways.

To further illustrate the relationship between topological properties and relevant biological functions, GO enrichment analysis and KEGG pathway analysis are performed on top 100 targets predicted by the RWRHE_GIP and RWRHE_E algorithm, respectively, shown in S11 Table. The results of the two algorithms do not include the GO term ‘Positive regulation of transcription from RNA polymerase II promoter’, the GO term ‘inflammatory response’ and the KEGG pathway ‘Pathways in cancer’. The GO term ‘Positive regulation of transcription from RNA polymerase II promoter’ have contribution to the pathogenesis of AD, which has been discussed above. Numerous studies have revealed the strong contribution of inflammation to AD pathogenesis, Aβ deposition in AD is related to inflammatory response [43–45]. Some cancer-related signaling pathways including FOXO, mTOR, SIRT1, HIF, oxidative stress, inflammation, and metabolism have important roles in regulating aging and AD [46]. The GIP similarity and the weights based on entropy are necessary in integrating networks, which could further display the entries related to AD.

**Fig 5:**
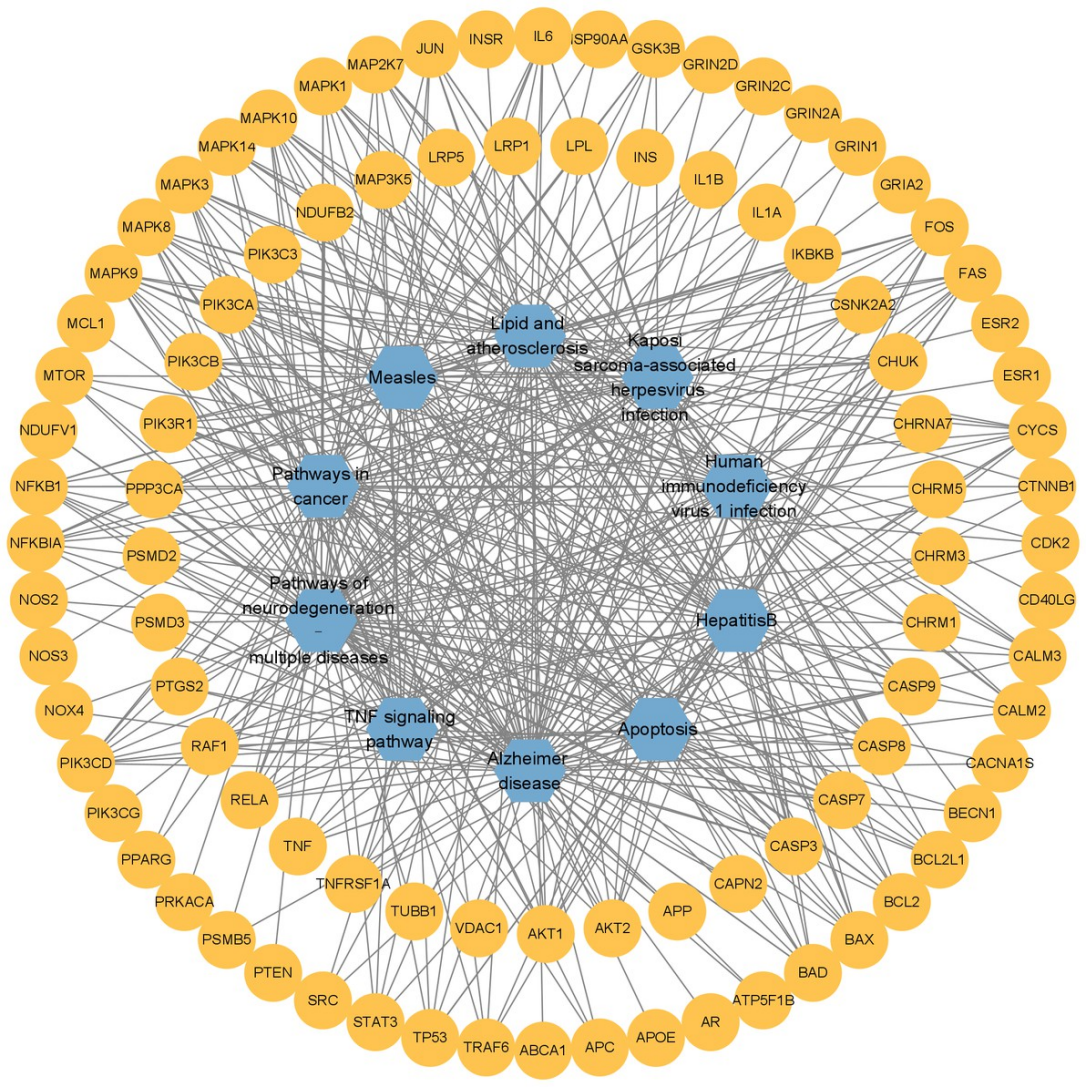
The pathway-target network. The orange circles represent targets, the blue hexagons represent pathways, and the gray lines represent the target-pathway connections.

To further explain the mechanism of multi-pathway and multi-target in the treatment of AD with TCMs, a target-pathway network is constructed based on the top 10 pathways of KEGG enrichment analysis. The relationships between the targets and the pathways are intuitively visualized, as shown in Fig 5. The same pathway is connected to different targets, in the meantime, the same target is connected to different pathways, which indicates that they are interactive relationships. Different targets play a synergistic role in the same pathway. And the pathway also regulates gene transcription and effects gene expression. Compared with single-target drugs, TCMs show the advantage of multi-target and multi-pathway.

### 3.4 Molecular docking

The components of TCM are complex and the targets are diverse. Molecular docking can indicate the binding ability between active ingredients and related targets. The Coach-D server is presented to predict the protein-ligand binding sites and ligand-binding poses by molecular docking [47]. The minimum value of Energy^u^ by Coach-D is regarded as the final docking energy to evaluate the binding ability between active ingredient and target.

The panel of ligands is composed of the top 15 active ingredients by RWRHE and several approved acetylcholinesterase (ACHE) inhibitors for AD. Several approved ACHE inhibitors for AD clinically are Galantamine, Donepezil, Rivastigmine, Tacrine and Huperzine A. The first four are approved by FDA, and Huperzine A is approved in China.

76 key enzymes and receptors from AD metabolic network [34] are sorted out to form the panel of proteins, and they are divided into 12 categories according to their main functions in the AD metabolic network, the structure versions of proteins are selected according to better resolution from Protein data bank (PDB), shown in S12 Table.

**Fig 6:**
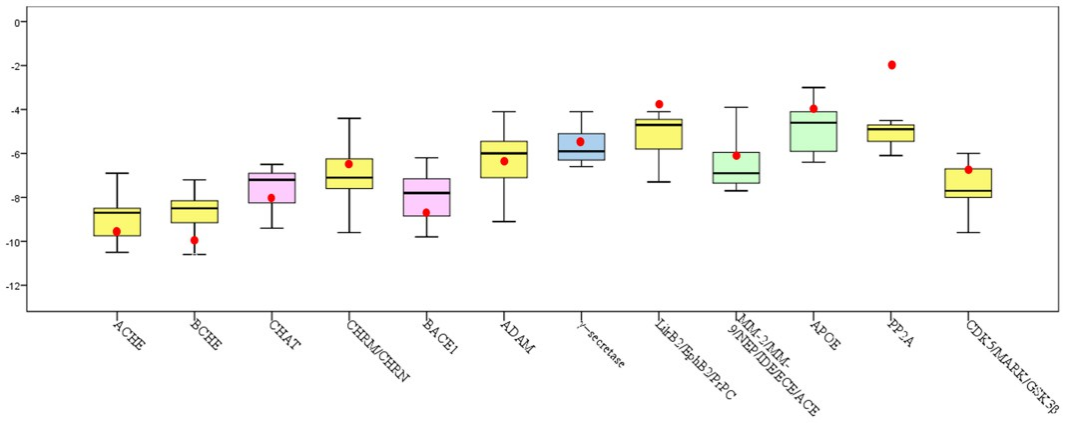
The box plot of molecular docking.

The minimum values of Energy^u^ for these possible complexes formed by ligands and proteins in the panels are calculated by Coach-D. The smaller the minimum value of docking energy is, the stronger the stability of active ingredient-protein site binding is. The docking energies of these five ACHE inhibitors and 12 categories of proteins are computed and the box plots are shown in Fig 6. The docking energies of these five ACHE inhibitors and their common target ACHE are used as the standard for active ingredients. The docking energies of the top 15 active ingredients and 76 proteins in the panel are given by Coach-D, then the average docking values of the top 15 active ingredients and 12 categories of proteins are computed and represented with red dots in Fig 6. The five approved ACHE inhibitors’ common target is the enzyme ACHE. BCHE is the isozyme of ACHE, the median docking energies of these five ACHE inhibitors with ACHE/BCHE are -8.7/-8.5, almost all docking values of the top 15 active ingredients and ACHE are less than -8.7 and the average value is -9.73, all docking values of the top 15 active ingredients and BCHE are less than -8.5 and the average value is -10.10. The results indicate that the top 15 active ingredients can bind to ACHE/BCHE and improve the level of acetylcholine (ACH), which is involved in both memory and learning. Similarily, the average docking value of the top 15 active ingredients and BACE1 is -9.45 and less than -8.7, the result shows that the top 15 active ingredients may bind to BACE1, which may reduce the generation of Amyloid-β (Aβ), Aβ is the main component of senile plaques. Among the top 15 active ingredients, some active ingredients are confirmed as ACHE or BACE1 inhibitors in previous research through wet experiments. Álvarez-Berbel et al. show that Quercetin and apigenin are characterized as inhibition of the enzymatic activity of ACHE [48]. Beg et al. report that the exposure of AD flies to kaempferol reduces ACHE activity [49]. In addition, Han et al. report that Baicalein exhibits strong BACE1 and ACHE inhibitory properties [50]. Youn et al. show that oleic acid exerts significant noncompetitive inhibitory activity against BACE1 [51]. Also, the docking results show that the top 15 active ingredients may bind to CDK5/MARK/GSK3β and restrain the phosphorylation of tau and reduce the formation of NFTs. It is well known that Memory loss, Senile plaques and NFTs are the main histopathological features of AD. The results show that the active ingredients can enter the active pocket of related targets well and act as multi-target agents in the prevention and treatment of AD.

## 4. Conclusion

AD is a complex neurodegenerative disease with few approved drugs. TCM has a long history and has a strong clinical basis for more than two thousand years. TCM has the advantage of more active ingredients, multi-target cooperative regulation and lower toxicity. Therefore, the study of new active ingredients from herbs is of great significance for the development of anti-AD drugs.

In this study, a novel random walk algorithm called RWRHE is devised to predict active ingredients and effective TCMs associated with AD based on entropy and random walk with the restart of a heterogeneous network. The comprehensive heterogeneous network is constructed by integrating the known active ingredient-target association network, active ingredient similarity network and target similarity networks based on entropy. The active ingredients and effective TCMs for AD are inferred based on random walks. The results measured by machine learning and bioinformatics show that the RWRHE algorithm achieves better prediction accuracy. Particularly, the docking energies of the five approved ACHE inhibitors and their common target ACHE are used as the standard for active ingredients, the results show that the top 15 active ingredients may improve the level of Ach, reduce the generation of Aβ and restrain the phosphorylation of tau, which are involved in the main histopathological features of AD. 20 herbs are ranked according to the active ingredients contained in herbs and ranks of active ingredients associated with AD, Danshen, Gouteng and Chaihu are recommended as effective TCMs for AD. Yiqitongyutang is recommended as effective compound for AD in the same way.

This study may provide new directions for building more effective prediction models to identify novel AD drugs. But there are several potential limitations in the current study that could be improved. In future, more validated association data would be incorporated to improve the quality of the heterogeneous network, and more useful and detailed studies would be integrated to improve the prediction ability. Only some predicted active ingredients have been validated for acting on key enzymes of AD in different published papers, we will take experimental validation into account and try to design wet experiments to verify the effectiveness of the predicted active ingredients in future.

## Supporting information

S1 Table TCM compounds-herbs-active ingredients. (XLSX)

S2 Table Active ingredients, targets and known active ingredient-target associations from TCMSP and HERB. (XLSX)

S3 Table The similarity values of active ingredients based on two similarity measures. (XLSX)

S4 Table The similarity values of targets based on four similarity measures. (XLSX)

S5 Table The comprehensive similarity values of active ingredients and targets. (XLSX)

S6 Table The final active ingredient-target association matrix *RT*. (XLSX)

S7 Table AD-target associations vector *P*. (XLSX)

S8 Table AD-active ingredient association vector *P*_*r*_. (XLSX)

S9 Table The positive and negative control groups. (XLSX)

S10 Table The predicted effect ranks of herbs in treating AD. (XLSX)

S11 Table GO enrichment analysis and KEGG pathway analysis on top 100 targets predicted by RWRHE_GIP and RWRHE_E. (XLSX)

S12 Table The panel of proteins. (XLSX)
